# Electrophoretic Coatings for Orthodontic Implants: Evaluation of Surface Properties, Adhesion, and Antibacterial Activity in Simulated Implantation Trials

**DOI:** 10.3390/jfb16090343

**Published:** 2025-09-12

**Authors:** Maria Biegun-Żurowska, Karolina Klesiewicz, Katarzyna Matysiak, Marcin Gajek, Alicja Rapacz-Kmita, Magdalena Ziąbka

**Affiliations:** 1 Department of Ceramics and Refractories, Faculty of Materials Science and Ceramics, AGH University of Krakow, 30 Mickiewicza Av., 30-059 Krakow, Poland; kmatysiak@agh.edu.pl (K.M.); mgajek@agh.edu.pl (M.G.); kmita@agh.edu.pl (A.R.-K.); 2 Department of Pharmaceutical Microbiology, Faculty of Pharmacy, Jagiellonian University Medical College, 9 Medyczna Str., 30-688 Krakow, Poland; karolina.klesiewicz@uj.edu.pl

**Keywords:** EPD, orthodontic implants, protective coatings, Ti6Al4V, chitosan, antibacterial properties

## Abstract

In this study, the properties of electrophoretically deposited (EPD) coatings on orthodontic implants made from Ti-6Al-4V alloy were evaluated during simulated implantation trials on animal bones. Three types of chitosan-based coatings were prepared using EPD: titanium nitride microparticles (TiNPs), titanium nitride nanoparticles (TiNNPs), and boron nitride particles (BNPs). Each of these coatings was also modified by adding a polylactic acid (PLA) layer using a dip-coating technique to compare their properties with and without this additional layer. The coatings were analysed using optical microscopy, confocal microscopy, and scanning electron microscopy (SEM) with elemental analysis. Surface roughness measurements of the coated implants were also conducted to highlight differences that could significantly influence the type and strength of the bone-implant interface, directly affecting the stability of the implant as an anchorage unit. Eventually, to evaluate the antibacterial properties of the EPD coatings, their antibacterial activity against both Gram-positive and Gram-negative bacteria strains was tested. Scanning electron observations confirmed the homogenous distribution of micro- and nanoparticles in all coatings. The highest surface roughness values were observed in layers containing titanium nitride nanoparticles (TiNNPs) and chitosan. The presence of an additional dip-coating PLA layer improved the adhesion, and its effect on the surface roughness depended on the particle size. While the antibacterial properties of the coatings show promising results, achieving optimal adhesion of the coatings to implants remains a challenge that requires further development.

## 1. Introduction

The use of implants and biomaterials in modern medicine is becoming increasingly prevalent due to advancements in these fields. However, this progress is largely driven by the growing demand for “spare parts” for the human body, a direct consequence of an aging population. Another significant factor is the shift in human lifestyles, which, combined with medical advancements, allow for improved quality of life. Implants play a crucial role in mitigating the negative effects of disabilities or even serving as components of aesthetic medicine.

In orthodontics, implants are widely utilized to enhance the quality of life for individuals requiring tooth stabilization, such as after injuries caused by accidents or to address aesthetic concerns. In many cases, the use of a temporary anchorage device (TAD) becomes necessary. TADs are temporarily fixed to the bone to enhance orthodontic anchorage by either supporting the teeth of the reactive unit or eliminating the need for a reactive unit altogether. Once their purpose is fulfilled, these devices are removed.

Even though Branemark et al. [1] observed in 1969 a strong anchorage of titanium in bone without adverse tissue reaction, the use of TADs was first reported in 1983, when Creekmore and Eklund [2] used a bone screw to treat a patient with a deep overbite. Costa et al. [3] developed a 6 mm long, 1.2 mm diameter titanium implant in 1999, and Sugawara and Umemori [4] introduced in 1999 the skeletal anchorage system (SAS) for anchorage in orthodontic treatment by using miniature titanium plates and monocortical screws that are temporarily fixed to achieve absolute anchorage. In 2001, Lee, Park, and Kyung [5] treated for the first time a case in which a micro-screw was used for orthodontic treatment. A micro-screw has three main components: a core, a helix (called a thread), and a head, which essentially serves two functions: it provides a means of applying torque to the core and thread, and it serves as a point of application of force. The core, which is the support of the screw, is attached to the head and wrapped in a screw thread. Micro-screws are most often made of metal using stainless steel, cobalt-chromium-molybdenum alloys or titanium and its alloys [6].

Although medical implants made of metals and their alloys are characterized by relatively high biocompatibility, their implantation into living tissues may result in adverse reactions [7,8], carrying the risk of postoperative complications, including the release of metal ions [9], the development microbial infections [8], excessive collagen deposition, and tissue necrosis in severe cases [9,10,11]. One well-established approach involves tailoring the implant surface through the application of multifunctional coating [11], which can serve simultaneously as an antibacterial barrier and as protection against corrosion and mechanical damage. These functional effects can be achieved by incorporating suitable antimicrobial agents—such as various nanoparticle types—into the coating composition [7,12]. Among available options, titanium nitride (TiN) and its derivatives stand out as highly promising for protective coatings owing to their exceptional hardness, outstanding resistance to wear and scratching, favourable surface wettability, low friction coefficient, and inert behavior in physiological conditions [13,14]. In addition, TiN demonstrates low toxicity in both in vitro and ex vivo conditions [14] and has been investigated for its potential antibacterial properties [15,16]. The deposition of TiN layers on implant devices has been shown to improve the biocompatibility and tribological characteristics of bone-contacting surfaces [17]. Moreover, particulate forms of TiN gain attention as alternative materials for a variety of biomedical applications [18,19].

Another promising candidate material for protective coatings is boron nitride (BN). Similarly to titanium nitride, BN is the compound that exhibits outstanding properties such as corrosion protection, mechanical and tribological properties including high hardness, excellent thermal stability, and low friction, making it suitable for various applications [20]. Boron nitride is also known for its biocompatibility and physiological inertness, which are crucial for its use in medical devices [21]. Research has been conducted to demonstrate its possible antibacterial activity, which may contribute to improving the effectiveness of implant coatings by lowering infection risk and supporting more efficient integration with surrounding tissue [22,23,24].

The use of BN in particulate form, similar to TiN, is increasingly being considered for biomedical applications, including dental and orthopaedic implants [22]. The unique properties of BN, combined with its potential to reduce friction and wear, make it an excellent candidate for the longevity and functionality of implants, offering an alternative or complement to titanium-based coatings.

The EPD method, which utilizes the migration of charged particles in solution under the influence of an electric field, ensuring their methodical deposition on a substrate, is currently gaining increasing interest as an effective solution for the processing of bioactive coatings and biomedical nanostructures [25] and seems to be ideally suited to the process of applying chitosan-based coatings, as it offers a number of advantages, including the possibility of simultaneous co-deposition of different materials [26], the possibility of performing the deposition process at room temperature [26], as well as the ability to adapt to complex substrate shapes.

Bacterial contamination remains a major challenge in implantology, as microbial colonization can lead to peri-implant infections, delayed healing, and even implant failure. Studies have shown that the failure rate of orthodontic TADs can be as high as 30%, with inflammation of the surrounding tissue being a contributing factor [27,28]. TADs are particularly susceptible to bacterial adhesion due to their exposure to the oral environment. Therefore, the development of implant coatings with antibacterial properties is crucial to reduce the risk of infection and improve clinical outcomes. The incorporation of bioactive agents such as titanium nitride (TiN) and boron nitride (BN) into chitosan-based coatings offers a promising approach to enhance both biocompatibility and antimicrobial efficacy [28].

The main objective of this study was to evaluate the coatings properties and investigate whether the addition of TiN and BN particles to chitosan-based coatings obtained by electrophoretic deposition (EPD) affects the antibacterial properties to these coatings, which can improve the properties of metallic implants in orthodontic applications. Furthermore, the influence of TiN and BN particles incorporated into chitosan-based coatings on their microstructure, chemical composition, and surface characteristics when applied to Ti-Al-V alloy was examined. A further objective of the study was to evaluate the effect of an additional PLA layer on the coating adhesion and long-term stability (over 30 days according to MDR regulations) during implantation experiments. An additional PLA layer was designed to provide sufficient mechanical stability without compromising ease of implant removal or causing bone trauma. By strategically enhancing selected surface parameters, the work addresses key factors in optimizing the performance of biomedical coatings, while also highlighting their potential antibacterial properties.

## 2. Materials and Methods

### 2.1. EPD Layer Manufacturing on Micro-Implants

Orthodontic Temporary Anchorage Devices (TADs) featuring a self-drilling thread of 1.4 mm in diameter were manufactured from Ti-6Al-4V alloy by Dentos (Dalseo-Gu, Daegu, Republic of Korea). The coatings were produced via the Electrophoretic Deposition (EPD) method, employing different types of particulate materials. Titanium nitride microparticles (TiNPs) with an average diameter below 3 µm and boron nitride particles (BNPs) of approximately 1 µm in size were supplied by Sigma-Aldrich (St. Louis, Germany). Furthermore, titanium nitride nanoparticles (TiNNPs) with an average size of 20 ± 5 nm were procured from Plasmachem GmbH (Berlin, Germany), with the information on particle size based on the data provided by the manufacturers. A detailed physicochemical characterization of all particles used in this study is provided in the Supplementary Materials (Figures S1–S5).

For each EPD process, the working suspension was obtained by combining two separately prepared precursor suspensions. The first was produced by dissolving 0.125 g of chitosan (molecular weight: 100,000–300,000; Thermo Scientific, Waltham, MA, USA) in 20 mL of distilled water and 0.9 mL of acetic acid (Pureland, Kraków, Poland). The second precursor consisted of TiNPs, BNPs, or TiNNPs dispersed in a solvent mixture of 5 mL isopropyl alcohol (Pureland, Kraków, Poland) and 25 mL ethanol (POCH, Gliwice, Poland). In the final EPD formulation, the particle concentration was adjusted to 0.5 wt%. Prior to mixing the two precursors, each was subjected to ultrasonic agitation for 1 h to ensure uniform dispersion. After mixing, the combined suspension was again ultrasonically mixed for another hour and then for 24 h using a magnetic stirring. Just prior to the deposition process, the final suspension was ultrasonically mixed for 2 min to ensure uniform dispersion.

Before applying EPD coatings, titanium alloy micro-implants were prepared through a multi-step cleaning and etching process. Initially, the micro-implants were ultrasonically cleaned in acetone (Pureland, Kraków, Poland) for 30 min, followed by a second ultrasonic treatment in ethanol (POCH, Gliwice, Poland) for another 30 min. The micro-implants were then etched in an aqueous solution of hydrofluoric acid (5%) (Chempur, Zgorzelec, Poland) for 30 s and carefully rinsed using distilled water.

The EPD process was conducted under specific voltage and time conditions adopted to the type of particles used. For TiN, deposition was performed at a voltage of 40 V for a duration of 1 min for BN, 15 V for 1 min; and for TiNNPs, 25 V for 25 s. During the deposition process, the Ti-6Al-4V alloy acted as the cathode, while the stainless-steel electrode served as the anode. A constant distance of 1 cm was kept between the electrodes for the entire duration of the process.

After the initial coating process, half of the samples from each coating group were covered with an additional protective layer of poly(lactic acid) (PLA). The PLA coating was prepared using a 10 wt% solution of PLA (Corbion, Amsterdam, The Netherlands) dissolved in dichloromethane (POCH, Gliwice, Poland). The coating was applied by dipping the samples into the solution with an Osilla dip coater (Leiden, The Netherlands), using a dip speed of 1 mm/s, a dwell time of 5 s, and a drying time of 15 s. The PLA layer was introduced to enhance the adhesion of TiN and BN coatings and to provide additional protection against environmental factors such as oxidation, mechanical wear, or chemical degradation.

**Figure 1:**
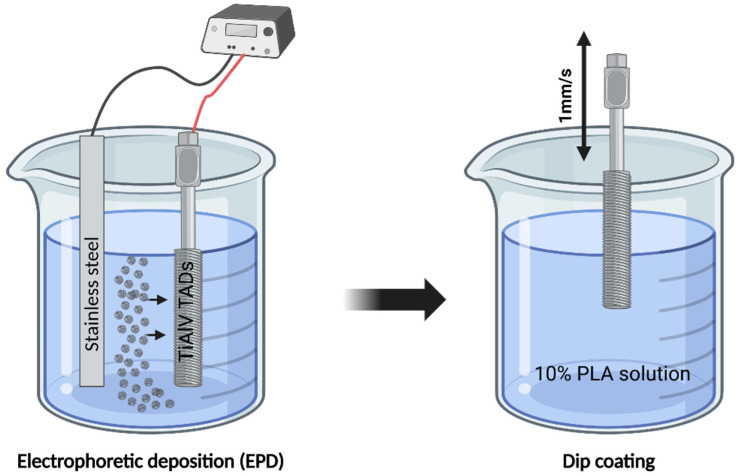
Schematic representation of the coating preparation process.

This comprehensive methodology ensured uniform coating application and effective preparation of the implants for further experimental evaluation. Samples of the 6 types of micro-implants were assigned to different groups according to each type of layer, as described in Table 1. The coating preparation process is presented in Figure 1.

### 2.2. Implantation of Micro-Implants into the Bone—Ex Vivo Studies

**Figure 2:**
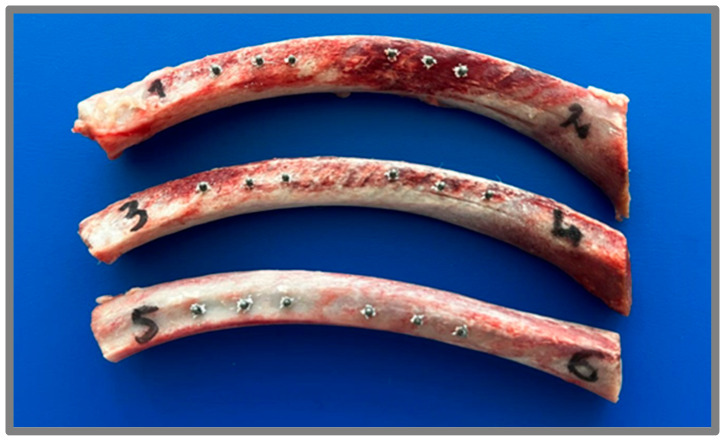
Rib bones used in ex vivo simulation after the orthodontic implantation.

Fresh pork rib bones, obtained from a local butchery, were used to evaluate the adhesion of the coatings under conditions simulating a clinical environment. As this study utilized post-mortem tissue and did not involve experimental animals, ethics committee approval was not required. The implants were screwed into the bone using a specialized dental mini-screw, following standard dental procedures and protocols. After the mechanical insertion test, the implants were removed and subjected to microscopic observations and further analyses to assess the integrity and adhesion of the applied coatings. The biological material after the implantation process is shown in Figure 2.

### 2.3. Material Examinations

#### 2.3.1. Optical Microscopy

Preliminary observation of the coated micro-implants before and after ex vivo simulation tests was performed using a Leica L9 stereoscopic microscope (Wetzlar, Germany) at ×5 and ×28 magnifications.

#### 2.3.2. Confocal Microscopy

The 3D topography was acquired with an Olympus LEXT OLS4000 confocal laser microscope (Olympus, Warsaw, Poland) using a 20× objective (FoV 640 µm × 640 µm). Measurements were taken at the flat, apical section of the implant thread, a surface that is functionally relevant during initial insertion. This specific region was selected for detailed analysis as it is both the most clinically susceptible to coating damage and its flat topography was essential for reliable, repeatable measurements.

Profile parameters Ra and Rz were computed according to ISO 21920-2:2021 [29] from 10 line profiles per specimen, each with an evaluation length of approximately 120–200 µm. A Gaussian L-filter was used with a cutoff wavelength (λc) of 80 µm and no S-filter was applied. The areal roughness parameters, Sa, Sz, were computed according to ISO 25178-2 [30] from the unfiltered height map of a nine 150 µm × 150 µm ROI after pointwise noise removal, with no S- or L-filtering and no plane or form removal.

#### 2.3.3. Scanning Electron Microscopy

The surface morphology and elemental composition of the coatings were analyzed using a scanning electron microscope (Apreo 2S Low Vac, ThermoFisher Scientific, Waltham, MA, USA) with an energy-dispersive X-ray spectroscopy detector (EDS; Octane Elite, EDAX, Tilburg, The Netherlands). Micrographs were acquired in low-vacuum mode (50 Pa) with a low-vacuum secondary electron detector (LVD) at an accelerating voltage of 5–10 kV at the flat, apical section of the implant thread. For elemental analysis, EDS area scanning was conducted under the same vacuum conditions, but at a voltage of 15 kV with a beam current of 1.6 nA. The acquisition time was 50 s, resulting in a detector deadtime of 30–40%. The EDS measurement was performed on the entire area of the visible LVD image at 5000× magnification.

#### 2.3.4. Antibacterial Activity

The reference bacterial strains applied in this study were sourced from the American Type Culture Collection (ATCC) and comprised the Gram-positive *Staphylococcus aureus* ATCC 25923 and the Gram-negative *Escherichia coli* ATCC 25922. The antimicrobial performance of the selected biomaterials (TiNPs, TiNNPs, BNPs) was assessed in accordance with the ASTM E 2180-07 guideline entitled *Standard Test Method for Determining the Activity of Incorporated Antimicrobial Agent(s) in Polymeric or Hydrophobic Materials* [31], incorporating minor procedural modifications.

In brief, each bacterial strain was cultured overnight in 50 mL of Tryptic Soy Broth (TSB); (BD BBL™, Franklin Lakes, NJ, USA). From these pure cultures, suspensions were prepared and introduced into an agar slurry composed of 0.7% Brain Heart Infusion (BHI) agar (Oxoid Ltd.; Basingstoke, UK) and 0.85% NaCl, yielding a final concentration of 5 × 10^5^ CFU/mL. Aliquots of 50 µL of the inoculated slurry were applied to the test specimens positioned in 24-well flat-bottom plates (NEST Biotechnology, Wuxi, China), allowed to solidify, and subsequently incubated at 37 °C for 24 h. Following incubation, each sample was transferred into a sterile container holding 2 mL of Brain Heart Infusion (BHI) broth (Oxoid Ltd.; Basingstoke, UK). To dislodge bacteria into the liquid medium, the specimens were subjected to 1 min of sonication followed by 1 min of vortexing at room temperature. The resulting bacterial suspensions were serially diluted (10^−1^ to 10^−6^) in fresh BHI broth using 2 mL Eppendorf tubes. From each dilution, 100 µL was spread onto Mueller–Hinton agar plates (Oxoid Ltd.; Basingstoke, UK) and incubated at 37 °C for 24 h. Colony counts were then performed, and CFU/mL values were calculated. All experiments were carried out in triplicate.

#### 2.3.5. Statistical Analysis

The statistical significance of differences in roughness parameters was determined using two separate two-way analyses of variance (2-way ANOVA), followed by Duncan’s post hoc tests. The first analysis assessed the effects of particle size and the PLA coating within the TiN-based groups (indicated by lowercase letters), while the second evaluated the effects of particle composition (TiN vs. BN) and the PLA coating (indicated by uppercase letters). All calculations were performed using Statistica 13.3 software (TIBCO Software Inc., Palo Alto, CA, USA) at a significance level of *p* < 0.05.

## 3. Results

### 3.1. Optical Microscopy

**Figure 3:**
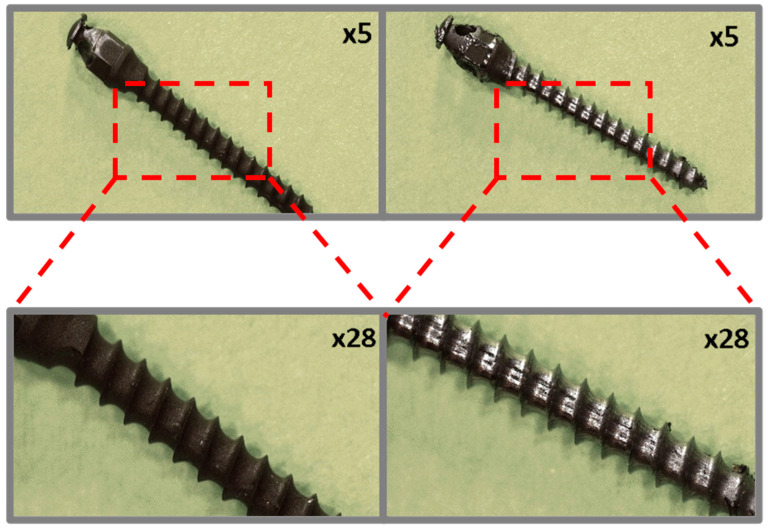
TiAlV orthodontic implants coated with TiNP layer before (**left**) and after (**right**) ex vivo simulation.

**Figure 4:**
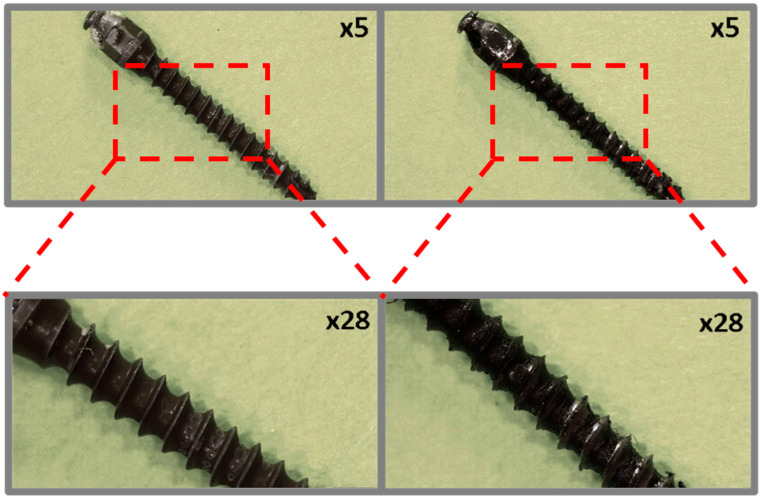
TiAlV orthodontic implants coated with TiNP/PLA layer before (**left**) and after (**right**) ex vivo simulation.

**Figure 5:**
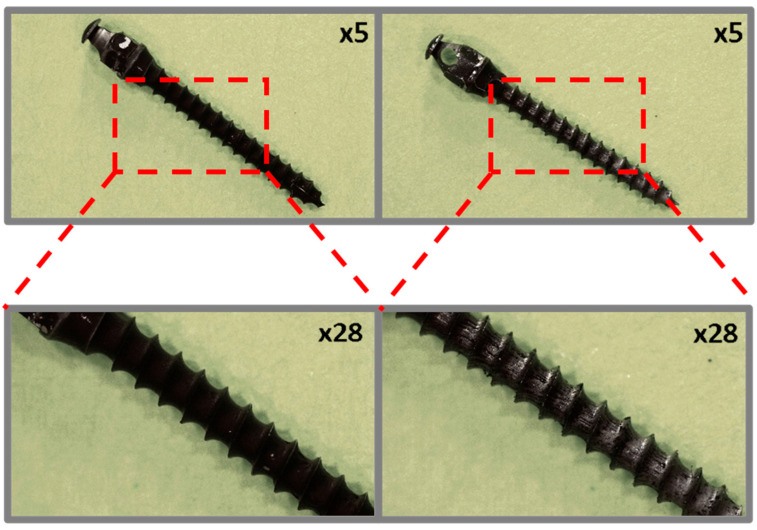
TiAlV orthodontic implants coated with TiNNP layer before (**left**) and after (**right**) ex vivo simulation.

**Figure 6:**
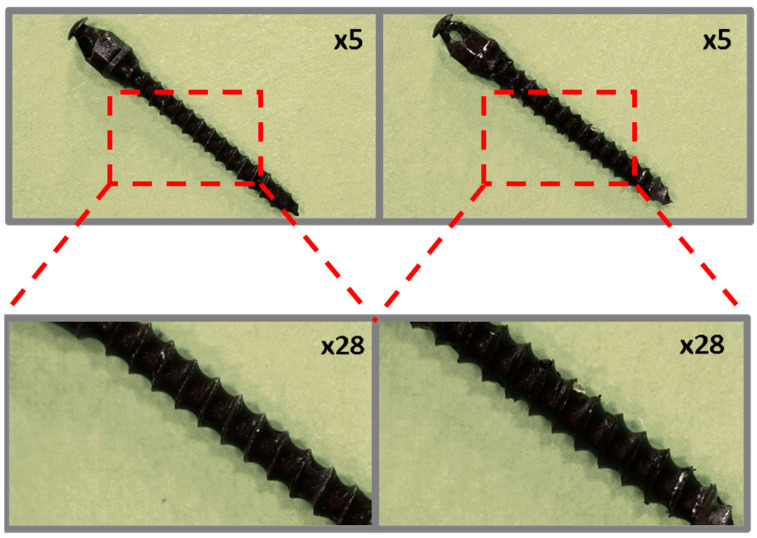
TiAlV orthodontic implants coated with TiNNP/PLA layer before (**left**) and after (**right**) ex vivo simulation.

Microscopic images of the coated micro-implants illustrating the comparative analysis of the coating condition before and after simulated ex vivo tests are shown in Figure 3, Figure 4, Figure 5 and Figure 6. The TAD micro-implant coated with a TiNP layer prior to the ex vivo simulation (Figure 3, left) exhibits a homogeneous coating with small, uncovered areas, primarily observed at the thread edges and the tip of the implant head. The absence of a coating on the head of the micro-implant is due to the fact that only the threaded part comes into direct contact with the bone, and the coating was applied exclusively to this section. In contrast, the analysis of the TAD implant after the ex vivo simulation (Figure 3, right) indicates that the adhesion of the coatings was insufficient to withstand the increased mechanical forces associated with implant placement in the rib bones. Only residual fragments of the damaged layer are visible on the implant threads.

In the case of the orthodontic micro-implant coated with a TiNP layer and an additional PLA layer, shown in Figure 4, the transparent PLA layer before the ex vivo simulations is visible at higher magnification only due to the presence of bubbles, located primarily in the spaces between the thread edges. Microscopic analysis of the TAD micro-implant after the ex vivo simulation test (Figure 4, right) confirmed that the additional PLA protective layer significantly improved the protection of the underlying EPD base layer. However, coating discontinuities after the ex vivo simulation tests were observed primarily at the thread edges and at the implant tip.

The coating containing smaller diameter TiN nanoparticles (TiNNPs) provided significantly improved initial uniformity, almost completely covering the implant threads with only minor discontinuities (Figure 5, left). However, the mechanical forces during implantation caused the coating to partially detach, leaving just a thin, uniform layer on the surface (Figure 5, right).

For the micro-implant coated with a TiNNP layer via EPD and an additional PLA layer (Figure 6), the EPD coating was uniformly deposited, and the PLA layer was much more visible, likely due to its greater thickness, particularly at the edges of the helix. The uneven thickness of the PLA layer resulted from the dip-coating process on a complex geometry. After ex vivo simulation (Figure 6, right), the use of smaller TiN particles combined with an additional PLA layer allowed the EPD layer to remain largely intact, with significant bone remnants visible on the implant surface.

**Figure 7:**
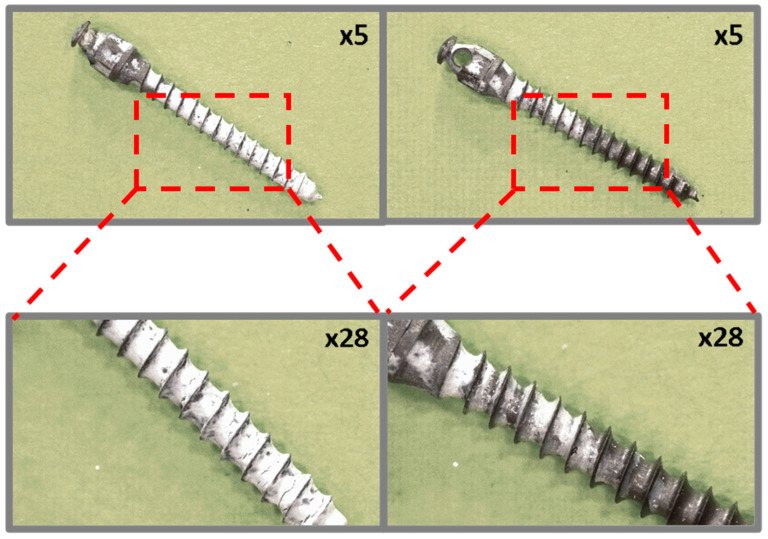
TiAlV orthodontic implants coated with BNP layer before (**left**) and after (**right**) ex vivo simulation.

Microscopy images of a TAD micro-implant coated with boron nitride particles, shown in Figure 7 illustrating its condition before and after ex vivo simulation testing, indicate that the original boron nitride coating is uneven, with incomplete coverage of the helix edges. Cracks in the layer are visible, likely caused by excessive EPD layer thickness (Figure 7, left). Microscopy images taken after the implantation simulation showed that the coating was not adequately bonded and resistant to the ex vivo test conditions, leaving only small residues on the micro-implant surface.

**Figure 8:**
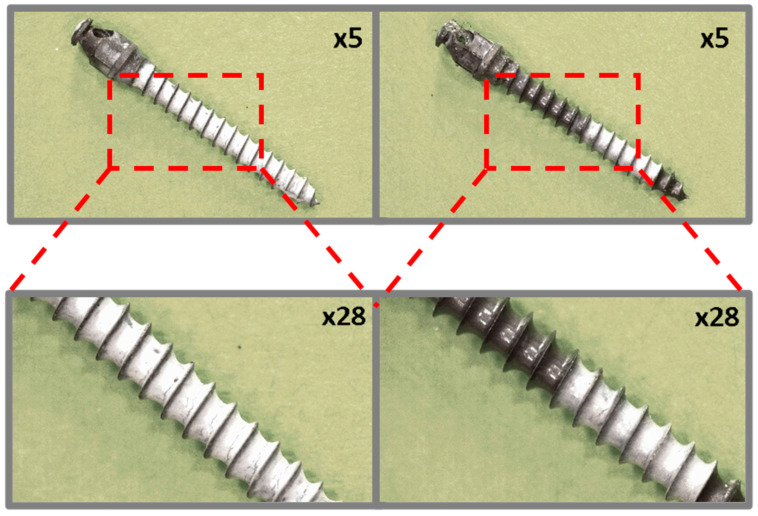
TiAlV orthodontic implants coated with BNP/PLA layer before (**left**) and after (**right**) ex vivo simulation.

Figure 8 shows microscopic images of a dental micro-implant coated with an EPD layer of boron nitride particles and an additional PLA layer, before and after ex vivo testing. These images clearly show that the original coating exhibits a predominantly uniform distribution across the implant surface, with incomplete coverage at the edges of the helix. Minor cracks are visible in the coating structure, and post-implant analysis revealed that the coating was almost completely degraded over most of the assessed surface area.

### 3.2. Confocal Microscopy

**Figure 9:**
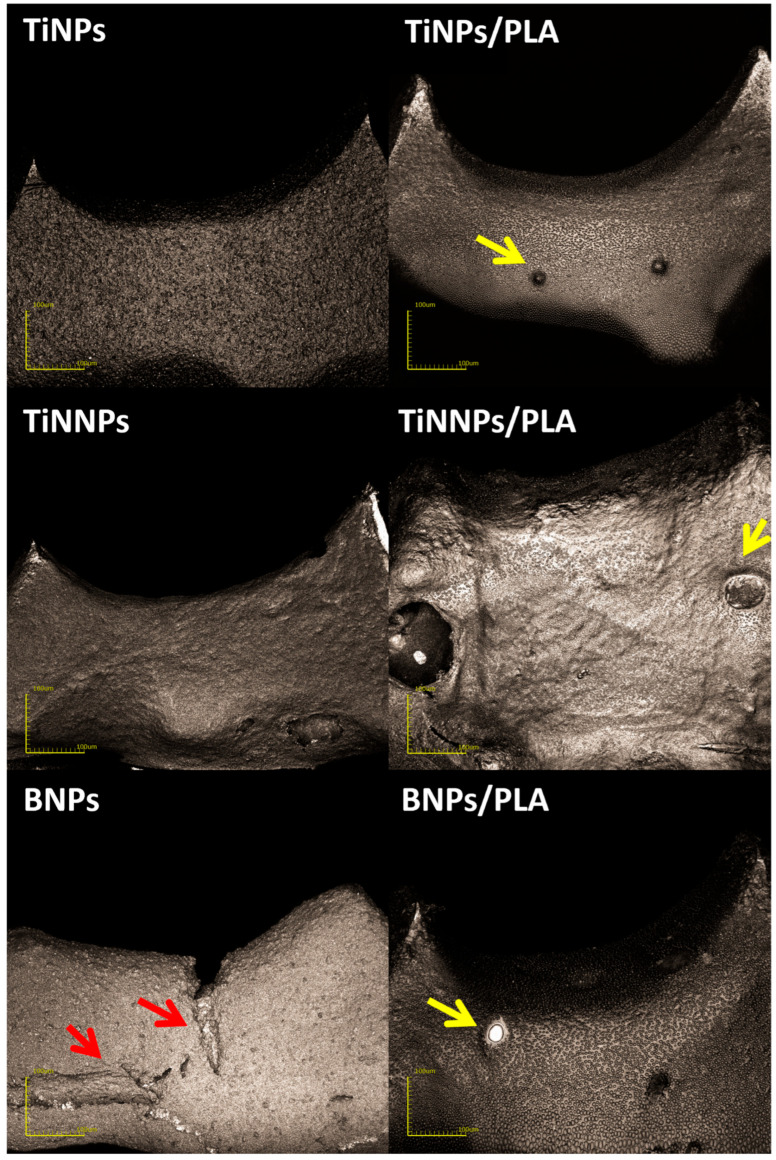
Three-dimensional confocal microscopy images of coated TiAlV orthodontic implants prior to ex vivo simulation testing. Red arrows indicate cracking observed in the boron nitride (BN) coating, while yellow arrows point to air bubbles entrapped under the protective PLA layer.

**Figure 10:**
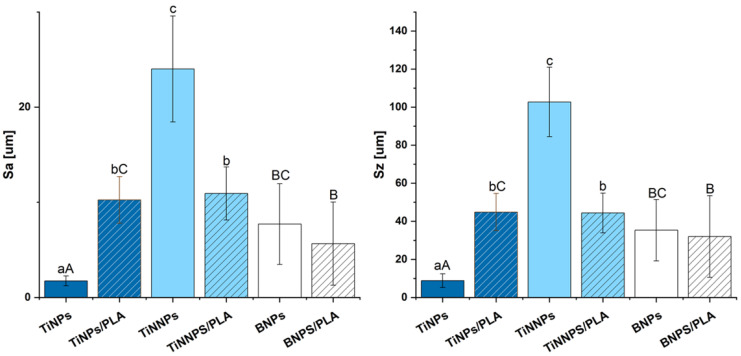
Surface roughness parameters, Sa and Sz, of coated implants before the ex vivo simulation tests. Statistical significance was determined by two-way ANOVA with Duncan’s post hoc test (*p* < 0.05). Lowercase letters (a–c) denote significant differences for the analysis of particle size and PLA coating within TiN-based groups. Uppercase letters (A–C) denote significant differences for the analysis of particle composition (TiN vs. BN).

Figure 9 displays three-dimensional confocal microscopy images of the coated Ti6Al4V orthodontic implants prior to the ex vivo simulation. The corresponding surface roughness parameters (Sa, Sz) are presented in Figure 10.

The micrographs focus on a critical part of the implant, the flat, apical section of the implant thread, which serves as the leading edge to facilitate implant insertion into the bone tissue. Images of implants coated with TiNP and TiNNP layers indicate uniform coverage of the helix tip, and comparative analysis of the layers revealed that larger TiNP particles were more visible on the surface. In contrast, as noted earlier, the implants with boron nitride (BN) coatings show significant cracking, as indicated by red arrows in the images. The deposition of a protective PLA layer via dip-coating provided complete surface coverage for all samples, while simultaneously altering the calculated surface roughness (Sa, Sz) and causing the occasional entrapment of single air bubbles on the surface confirm. (marked by yellow arrows in the images).

Notably, for the largest diameter particles, i.e., those in the titanium nitride (TiNP) layer, the Sa parameter increased nearly fivefold after the application of the PLA coating. Conversely, a reduction in surface roughness was observed for coatings containing smaller TiNNPs and boron nitride particles (BNPs). The reduction in surface roughness observed in BN-coated layers may be associated with the filling of cracks present in the BN layer by the PLA coating; however, this decrease was not statistically significant. This suggests that the interaction between the PLA layer and the underlying coatings varies with particle size and distribution, potentially influencing the mechanical and biological properties of the implant. These findings highlight the importance of optimizing coating systems for orthodontic applications, as the structural integrity, homogeneity, and surface roughness of the protective layers play a crucial role in ensuring implant performance and durability during surgical procedures.

**Figure 11:**
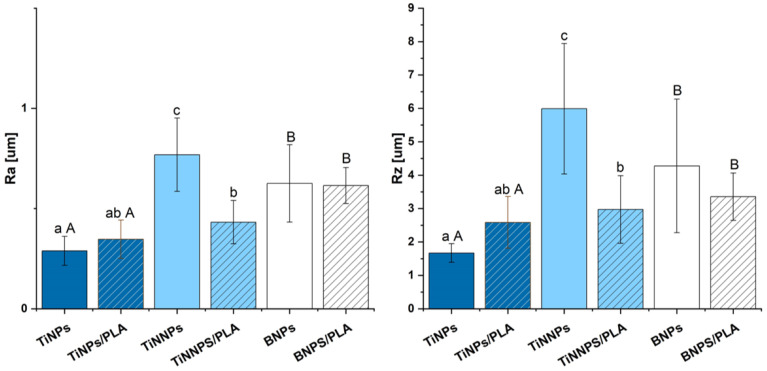
Linear roughness parameters, Ra and Rz, of coated implants before the ex vivo simulation tests. Statistical significance was determined by two-way ANOVA with Duncan’s post hoc test (*p* < 0.05). Lowercase letters (a–c) denote significant differences for the analysis of particle size and PLA coating within TiN-based groups. Uppercase letters (A, B) denote significant differences for the analysis of particle composition (TiN vs. BN).

To complement the area roughness analysis, linear roughness parameters were measured at the same critical section of the implant thread, and the results presented in Figure 11 are consistent with the previously discussed surface roughness parameters, showing similar trends. Among the tested samples, the coating containing the smallest nanoparticles without an additional PLA layer (TiNNPs) was characterized by the highest surface roughness (Ra, Rz), while the lowest roughness was measured for the TiNP coating.

**Figure 12:**
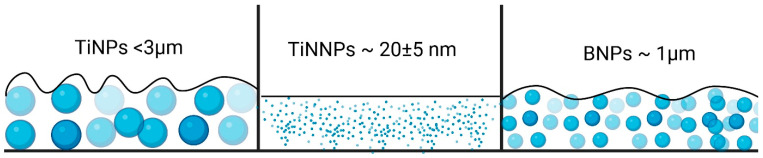
Graphical representation of the effect of the PLA layer on the surface roughness of TiNPs, TiNNPs and BNPs EPD coatings.

The potential impact of a PLA coating applied on an EPD layer with different particle sizes by dip-coating is graphically illustrated in the schematic diagram in Figure 12, which demonstrates the effect of particle size on surface roughness.

The influence of a subsequent PLA coating on the surface roughness of EPD layers was found to be dependent on the initial particle size, and for coatings containing larger TiNPs particles, the application of a PLA layer led to a slight increase in the mean roughness value; however, this change was not statistically significant (Figure 11).

A different trend was observed for coatings with smaller particles, where the PLA layer generally acted as a smoothing agent, which resulted in a statistically significant decrease in surface roughness for the nanoscale TiNNP (~20 ± 5 nm) coating. For the BNPs, a similar decrease in the mean roughness value was observed, although in this case, the change was not statistically significant.

This can be attributed to the PLA layer’s ability to fill voids and planarize the surface, with the effect being dependent on the initial particle topography. The most pronounced smoothing was observed for the layers containing TiNNP nanoparticles, where the polymer effectively filled the interstitial gaps, and a similar, though less impactful, mechanism likely occurred for the layers containing BNPs. Conversely, for the very large TiNPs, the polymer coating was insufficient to fully cover the significant initial surface irregularities, resulting in no significant change in roughness.

For temporary anchorage devices (TADs), surface roughness plays a pivotal role in establishing immediate mechanical stability, and a precisely engineered topography serves to maximize frictional resistance and mechanical interlocking with the surrounding bone tissue, thereby enhancing primary retention. This is essential for applications requiring stable anchorage without inducing osseointegration, thus facilitating subsequent device removal [32,33,34].

### 3.3. Scanning Electron Microscopy

**Figure 13:**
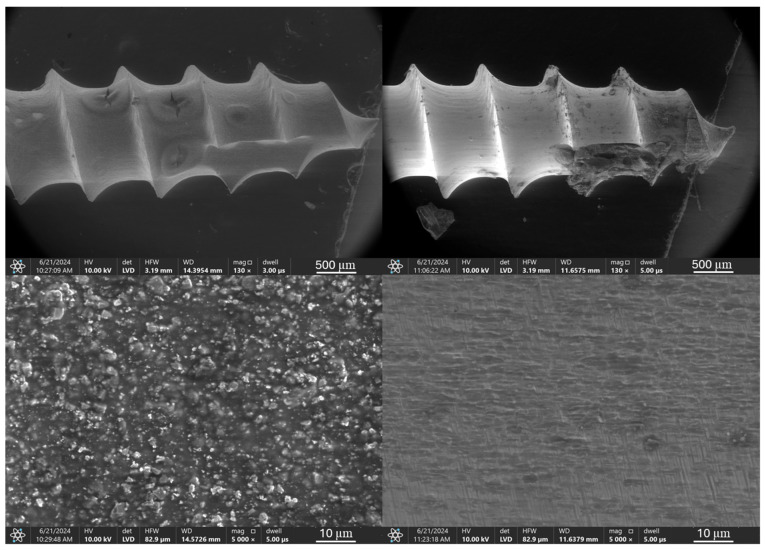

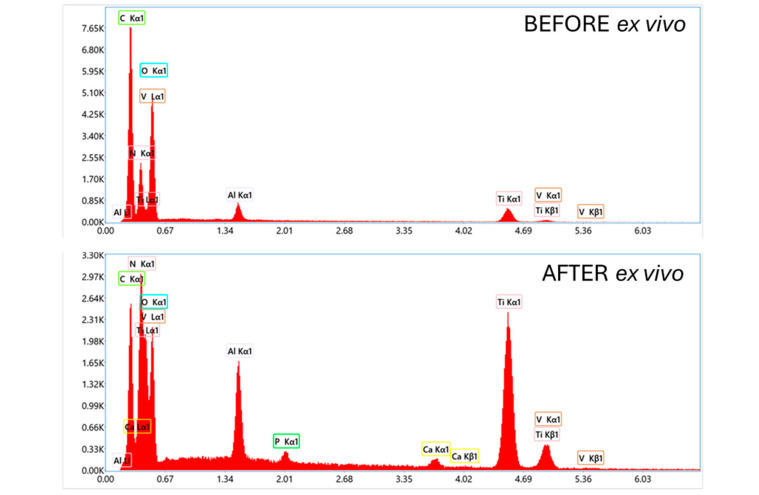
SEM images and EDS spectra of elemental contents in TiAlV orthodontic implant coated with TiNP layer before (SEM **left**, EDS **top**) and after ex vivo simulation test (SEM **right**, EDS **bottom**).

**Figure 14:**
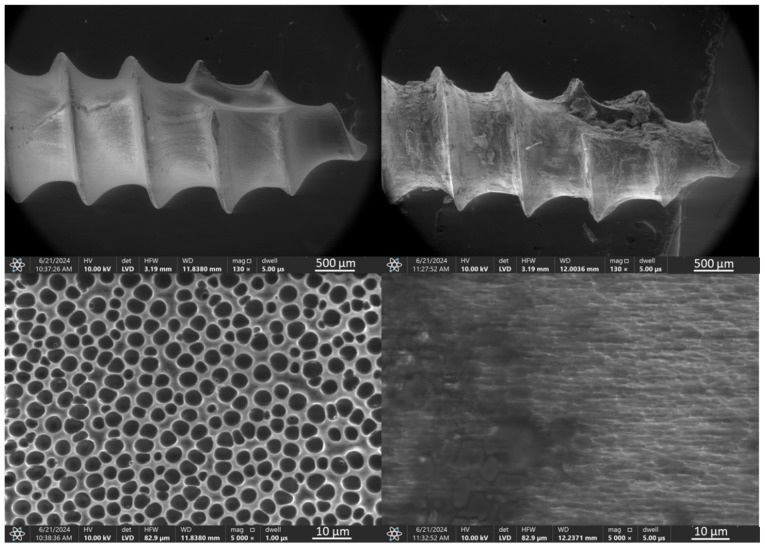

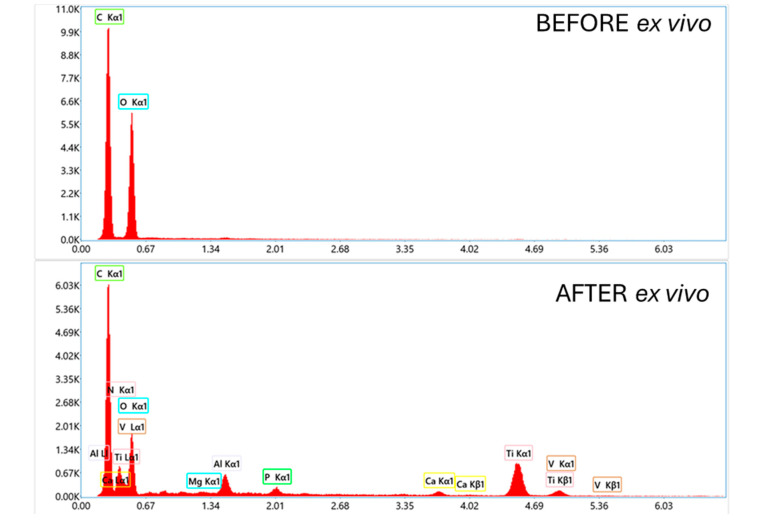
SEM images and EDS spectra of elemental contents in TiAlV orthodontic implant coated with TiNP/PLA layer before (SEM **left**, EDS **top**) and after ex vivo simulation test (SEM **right**, EDS **bottom**).

**Figure 15:**
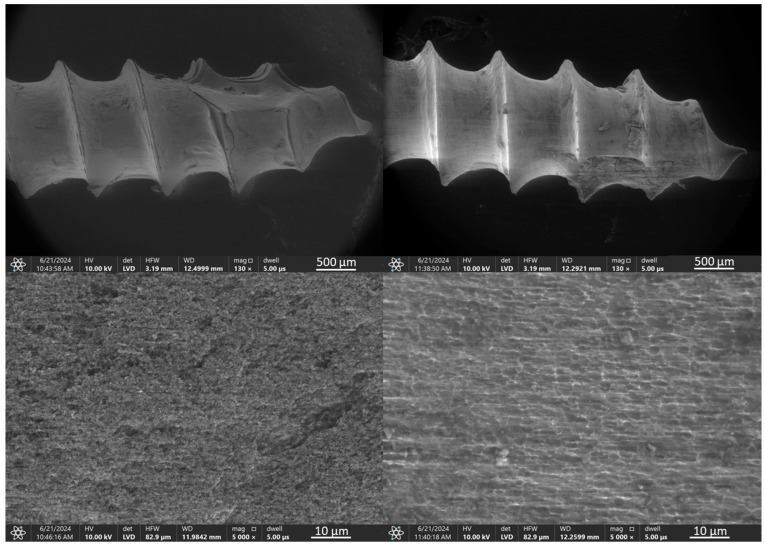

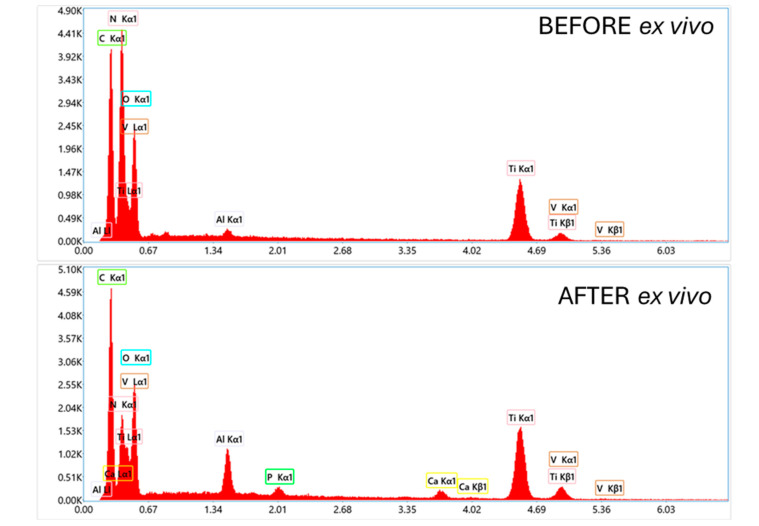
SEM images and EDS spectra of elemental contents in TiAlV orthodontic implant coated with TiNNP layer before (SEM **left**, EDS **top**) and after ex vivo simulation test (SEM **right**, EDS **bottom**).

**Figure 16:**
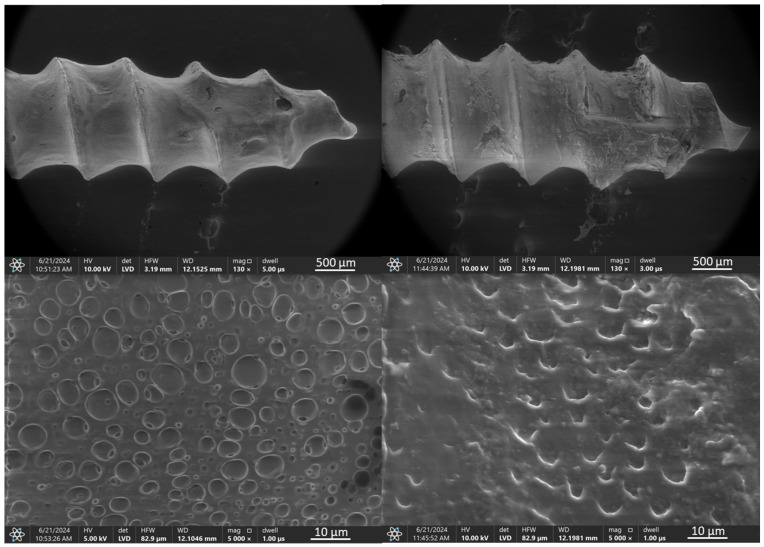

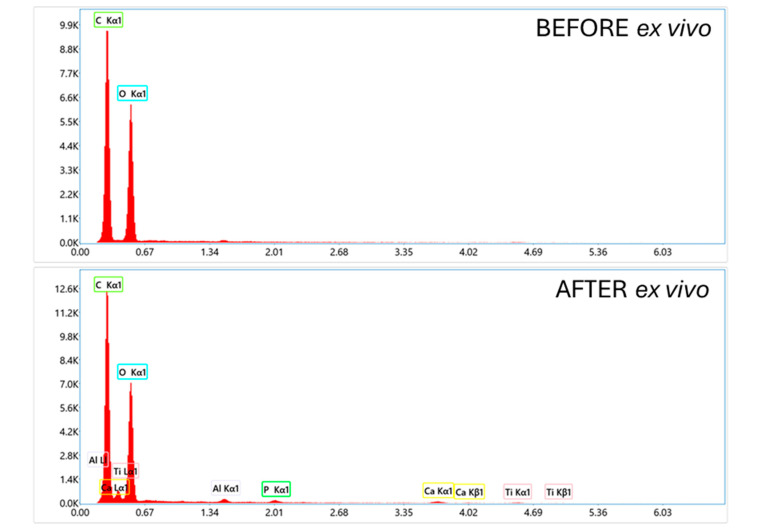
SEM images and EDS spectra of elemental contents in TiAlV orthodontic implant coated with TiNNP/PLA layer before (SEM **left**, EDS **top**) and after ex vivo simulation test (SEM **right**, EDS **bottom**).

**Figure 17:**
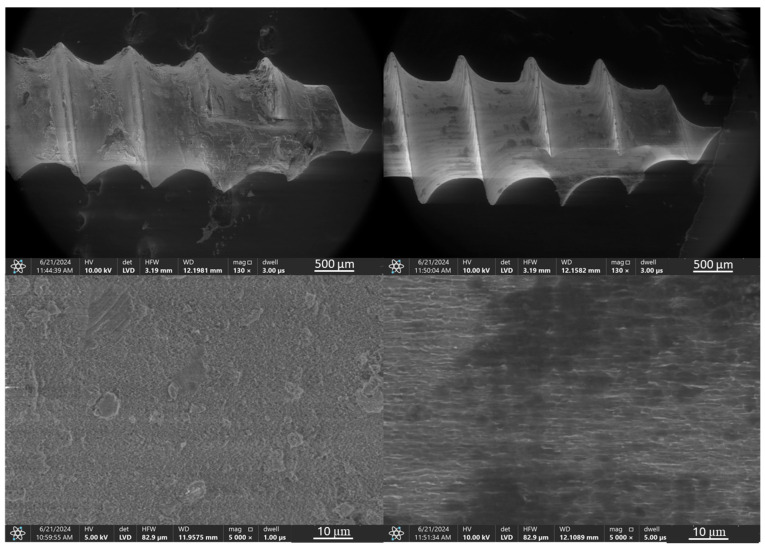

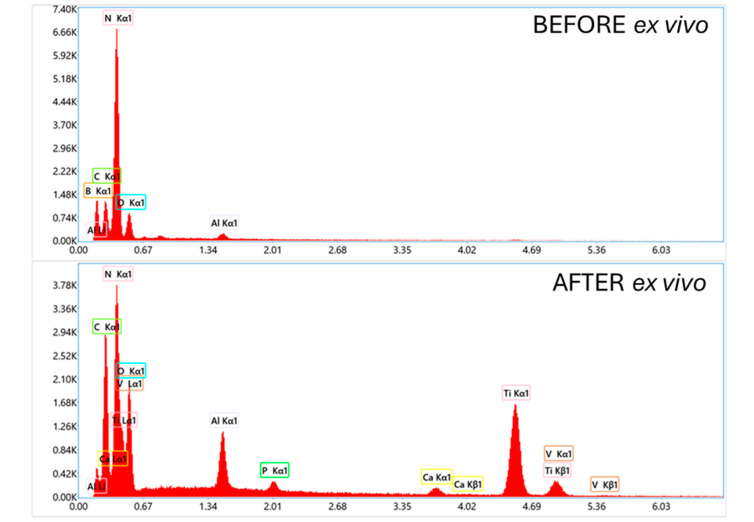
SEM images and EDS spectra of elemental contents in TiAlV orthodontic implant coated with BNP layer before (SEM **left**, EDS **top**) and after ex vivo simulation test (SEM **right**, EDS **bottom**).

**Figure 18:**
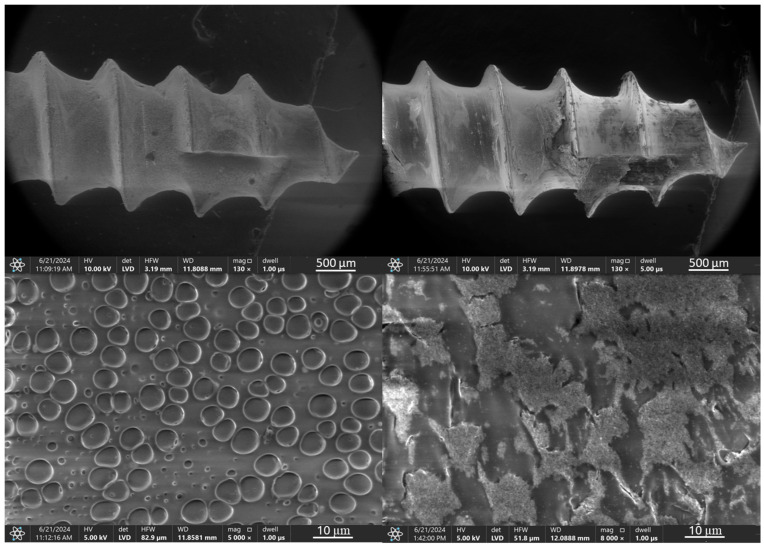

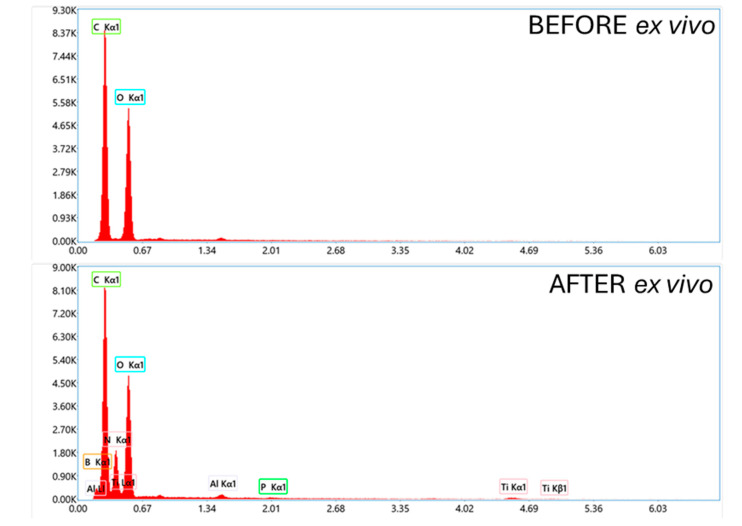
SEM images and EDS spectra of elemental contents in TiAlV orthodontic implant coated with BNP/PLA layer before (SEM **left**, EDS **top**) and after ex vivo simulation test (SEM **right**, EDS **bottom**).

A detailed analysis of the structure of the deposited coating systems and their composition both before and after the implantation tests was performed by scanning electron microscopy (SEM) with area energy dispersive spectroscopy analysis (EDS). To ensure consistency, the analysis focused on the same critical implant region previously selected for topographic characterization. The results are presented in Figure 13, Figure 14, Figure 15, Figure 16, Figure 17 and Figure 18.

SEM images of a TiNP-coated implant, shown in Figure 13, confirms that the initial coating completely covers the TAD implant, with only a few minor inhomogeneities. At higher magnification, uniform deposition of TiNPs is visible, and EDS analysis confirmed the implant substrate composition as titanium, aluminum, and vanadium, with the presence of nitrogen and carbon peaks attributed to the chitosan and titanium nitride EPD coating. After testing in the simulated ex vivo environment, SEM observations revealed damage to the coating, exposing the underlying implant surface, with minimal coating residue and contamination containing calcium and phosphorus, suggesting bone origin. Contaminants and coating residues were found primarily at the tip of the thread, near the leading edge of the implant.

The results of SEM observations with EDS analysis of a titanium nitride particle coating with an additional TiNP/PLA polylactic acid layer (Figure 14), reveal that prior to ex vivo testing, the coating is homogeneous and completely covers the implant. However, porous microstructure with pore size app. 5 µm was observed at higher magnifications, which is attributed to dichloromethane evaporation during PLA layer preparation. EDS analysis of the coating confirmed that the outer layer is composed primarily of carbon and oxygen, indicating the presence of PLA, and uniformly covers the underlying EPD layer. These results underscore the effectiveness of the PLA layer in encapsulating the TiNP coating, although images taken after the ex vivo testing revealed the presence of discontinuities in the layer as well as the presence of calcium and phosphorus derived from bone. EDS analysis revealed peaks for titanium, aluminum, and vanadium, consistent with the substrate composition, and additionally detected presence of calcium, phosphorus, and magnesium, suggesting bone origin. At higher magnifications, partial damage to the PLA layer was observed, exposing both the underlying EPD layer and the substrate.

SEM observations of the TiNNP coating before and after ex vivo simulations (Figure 15), demonstrate that before the simulations, the coating uniformly covered the implant, with nanoparticles evenly deposited and densely packed. After the ex vivo simulations, SEM analysis revealed degradation of the coating, with coating residues, along with bone debris, accumulating primarily at the implant tip. EDS analysis confirmed that the bone debris contained calcium, phosphorus, and magnesium, further indicating its origin from bone tissue.

The SEM observation results for the orthodontic implant with TiNNP EPD coating and an additional PLA layer shown in Figure 16, indicate that the coating system uniformly covers the entire implant, with the previously mentioned blisters visible at higher magnifications. Observations of the implant after the ex vivo test indicate that the coating generally withstood bone placement well, with only minor deformation of the outer PLA coating layer. However, EDS analysis detected peaks associated with the presence of aluminum and titanium, which may indicate a thin outer coating thickness or discontinuities. Additionally, peaks associated with bone-derived contaminants containing calcium and phosphorus were also identified.

The results of SEM observation and EDS analysis of an orthodontic implant coated with an EPD coating containing BNPs, shown in Figure 17, demonstrate that the coating completely covers the implant, but its structure is not uniform, exhibiting variations in thickness and cracks. At higher magnifications, deposition of BN particles is observed, with occasional agglomeration, and the results after the implantation test revealed complete damage to the coating, exposing the implant substrate. Here, too, additional bone-derived contamination was identified.

SEM analysis with EPD of a TAD implant coated with an EPD layer of BN particles and an additional PLA layer, shown in Figure 18, showed that prior to ex vivo simulation, the coating exhibited complete and uniform coverage of the orthodontic component. However, higher magnification revealed the presence of surface blisters, and EDS analysis confirmed the integrity and continuity of the outer PLA layer. Post-implant observations indicated partial degradation of the coating, and EDS analysis detected bone-derived debris and confirmed substrate exposure.

### 3.4. Antibacterial Activity

A summary of the quantitative results from antibacterial activity tests is presented in Table 2. These results compare the viability of bacteria on individual EPD layers, which differed in the types of particles used. Layers coated with an additional PLA film were not tested due to their proven bacterial neutrality [35,36]. For reference, bacterial survival on a pure Ti6Al4V alloy substrate was also evaluated.

In the case of Gram-positive bacteria, a significant reduction in the number of bacteria was observed for the TiNP coating, amounting to as much as 3 logarithmic units. In the case of the coating containing TiNNP particles, the reduction was 2 logarithmic units compared to the pure substrate. The weakest antibacterial activity was recorded for the coatings containing boron nitride particles. In the case of Gram-negative bacteria, a similar reduction was observed for the TiNP coating, with a decrease of 3 logarithmic units; however, no significant antibacterial effect of the TiNNP coating was noted against *E. coli* bacteria. The lower efficacy of the EPD coatings against Gram-negative bacteria, compared to Gram-positive bacteria, can be attributed to the distinct structural differences in their cell envelopes. Gram-negative bacteria possess an outer membrane, rich in lipopolysaccharides, which acts as an additional protective barrier external to the peptidoglycan layer. This outer membrane can impede the interaction of the antibacterial particles with the bacterial cell, thereby reducing their antibacterial efficacy. In contrast, Gram-positive bacteria lack this outer membrane, making their cell walls more accessible to the direct action of the antibacterial agents and consequently leading to a more pronounced antibacterial effect. This structural feature of Gram-negative bacteria likely contributes to the observed variations in antibacterial activity [37,38,39].

## 4. Discussion

The selection of research methods allowed to obtain results that provide arguments to confirm the thesis that the applied electrophoretic coatings (EPD) improve both the surface roughness and antibacterial properties of orthodontic implants, which perfectly corresponds with previous reports emphasizing the role of surface modification in improving implant mechanical anchoring and biocompatibility [40,41].

The orthodontic micro-implants used as the substrate for the coatings in our study were commercially available Temporary Anchorage Devices (TADs) made of a Ti6Al4V alloy. The characteristics of these implants prior to any modification have been documented in previous studies. In line with the findings of Sycińska-Dziarnowska et al. [42,43], the surface of an untreated TAD implant is relatively smooth, with a measured average roughness (Ra) of 0.208 ± 0.03 µm. Microstructural analysis of this baseline surface shows only minor embossing that results from the manufacturing process.

A key preparation step, both in our study and the cited literature, was etching with hydrofluoric acid (HF), a process intended to increase surface roughness for better layer adhesion and improved primary mechanical stability. The effects of this process on the bare implant surface were analysed in detail within the prior works. This treatment significantly increased the average roughness (Ra) up to 0.313 ± 0.06 µm, while exposing the underlying microstructure of the alloy and creating a more pronounced surface. Chemical analysis also detected the presence of residual fluoride on the implant surface following the etching process. Interestingly, despite the increase in roughness, the etched implants demonstrated one of the lowest levels of bacterial biofilm formation, comparable to the smooth, un-etched control implants.

Regarding its inherent antimicrobial capacity, the unmodified Ti6Al4V material was also evaluated in the cited literature [42], serving as a control group in biofilm formation assays against bacterial strains such as *S. aureus*, *E. coli*, and *S. mutans*. The results indicated that while these surfaces do allow for bacterial colonization, the standard Ti6Al4V implants demonstrated some of the lowest levels of biofilm accumulation when compared to several of the experimentally modified surfaces. This establishes that the base material has a degree of natural resistance to extensive biofilm formation but also highlights the potential for significant improvement through the application of advanced antimicrobial coatings.

In our study, analysis of the tested coatings on TAD implants revealed that the surface modification with TiNNPs yielded the highest roughness value (Ra = 0.769 ± 0.18 µm). While the direct clinical significance of this specific level of roughness requires further investigation, creating a more complex surface topography is a recognized strategy for potentially enhancing the initial mechanical retention of these devices [33,44]. However, the impact of surface roughness on coating adhesion is complex and varies depending on material composition and coating type. According to Zivic et al., increased surface roughness can lead to decreased coating adhesion, as rough surfaces create stress concentrations that may initiate cracks, leading to delamination and premature failure [45]. Nevertheless, other studies suggest that moderate roughness can enhance adhesion for specific coatings, highlighting the importance of optimizing surface morphology based on material-specific interactions [46].

Various studies also indicate that increasing surface roughness can improve the antibacterial properties of coatings. For example, fluorinated diamond-like carbon (F-DLC) coatings with higher roughness demonstrated enhanced antibacterial activity against *Escherichia coli*, eliminating up to 70% of the bacteria [47]. Similarly, copper coatings produced at higher sputtering currents, which resulted in increased surface roughness, demonstrated significant antibacterial efficacy against *Staphylococcus aureus* [48]. These findings suggest that controlled surface roughness modifications can play a crucial role in improving the antibacterial properties of implant coatings.

The antibacterial efficacy test results conducted in this study confirmed a significant reduction in bacterial viability for TiN-based coatings, confirming previous reports attributing the antimicrobial activity of TiN to its chemical stability and surface properties [49]. The TiNP coating demonstrated the highest antibacterial activity, particularly against Gram-positive *S. aureus*, and significantly reduced the viability of these bacteria as well as *E. coli* bacteria compared to the control group.

The TiNNP coating, for which the highest roughness was measured, demonstrated moderate antibacterial activity, demonstrating relatively good activity against *S. aureus* but only weak efficacy against *E. coli*, which may be due to differences in the cell wall structure of Gram-negative bacteria, which have an additional outer membrane that may limit interactions with TiNNPs. The BNP coating demonstrated the weakest antibacterial activity, with bacterial growth rates similar to those observed in the Ti6Al4V control group. Although BN layer has favorable biocompatibility and mechanical properties, its antibacterial activity may be insufficient to prevent bacterial colonization in orthodontic applications. In summary, it can be stated that TiN-based coatings may prove to be a promising solution for use in the strategy of reducing bacterial adhesion; however, the evaluation of the long-term antibacterial effectiveness of these coatings would require further research.

Despite promising antibacterial and proper mechanical properties, the durability of the tested coatings under implant conditions remains a challenge, with ex vivo test results indicating partial detachment of all coatings, which is also associated with the implant being twisted out of the bone and the application of force in the opposite direction. Such mechanical movement and opposite rotation definitely influence the deformation of the coating adhesion. Coating implants with dip-coated polylactic acid (PLA) layers improved adhesion of all particle types, consistent with the stabilizing properties of PLA reported in the literature [50,51]. Interestingly, while the PLA layer increased the roughness for larger particles, potentially fostering stronger bone-implant interfaces [52], it decreased the roughness of smaller particles, which may help minimize bacterial adhesion [53].

The TiNNP/PLA combination demonstrated the highest retention, supporting the hypothesis that nanoparticle size and secondary coatings are critical for increasing the layer resilience. These results are consistent with studies emphasizing the role of optimized nanoparticle distribution and additional layers in mitigating mechanical damage during implantation.

Finally, it is crucial to highlight a key interpretational aspect of this study concerning the role of particle size. The experimental design, focused on comparing materials that inherently differ in both chemical composition and physical dimensions, precludes a definitive isolation of size-dependent effects. While a thorough characterization of all particles was conducted, any observed variance in outcomes could be a result of the material’s intrinsic properties, its dimensions, or a synergistic effect of both. Therefore, the role of particle size has been presented throughout this discussion as a contributing factor within a complex system. A definitive quantification of its singular impact would necessitate a future experimental matrix where material composition is held constant while particle size is systematically varied.

In conclusion, this study underscores the need for an integrated approach to coating design, balancing surface roughness, antibacterial efficacy, and mechanical stability. Future research should explore advanced EPD techniques, such as multi-material layering, and evaluate the long-term performance of these coatings in vivo. Incorporating bioactive additives and optimizing the deposition parameters could further enhance the clinical efficacy of orthodontic implants, thereby reducing the risk of complications and improving patient outcomes.

## 5. Conclusions

In this study, six different coating systems consisting of an electrophoretic deposition (EPD) layer of chitosan combined with three different particle types, with and without an additional polylactic acid (PLA) layer, were tested on orthodontic micro-implants. Results indicated that coating adhesion was a key issue under the high mechanical stresses associated with implantation, with coatings containing smaller particles exhibiting superior adhesion and durability compared to coatings containing larger particles.

To address this, an additional PLA protective layer was deposited on all EPD coating types via dip-coating, and it was confirmed that this layer significantly improved the protection of the underlying EPD base layer. The PLA layer acted as a protective barrier, reducing detachment and improving the overall performance of the coating system and improving short-term mechanical stability between the bone and the implant; however, some discontinuities in the coatings were observed after ex vivo simulation tests, primarily at the thread edges and at the tip of the implants.

Additionally, the application of a protective PLA layer was found to affect the surface roughness of the implants, with the degree of roughness varying with the type of particles used in the EPD layer. This variability in surface characteristics suggests that the selection of particle type and the addition of PLA can be tailored to optimize implant properties for specific applications.

The study demonstrated that the antibacterial effectiveness of EPD coatings is strongly influenced by the type of particles used, with TiNP coatings showing the highest activity against both Gram-positive and Gram-negative bacteria. In contrast, boron nitride coatings exhibited the weakest antibacterial properties, while the TiNNP coating was primarily effective against Gram-positive bacteria but had no significant activity against *E. coli*.

These observations underscore the importance of optimizing particle size, surface roughness, and incorporating protective layers, such as PLA, to improve the adhesion, durability, and functionality of coatings for orthodontic implants operating under challenging conditions.

## Figures and Tables

**Table 1:** Sample nomenclature.

TiNPs	Orthodontic titanium alloy (TiAlV) implant covered with EPD coating containing chitosan and Titanium Nitride particles
TiNPs/PLA	Orthodontic titanium alloy (TiAlV) implant covered with EPD coating containing chitosan and Titanium Nitride particles with additional PLA protective layer
TiNNPs	Orthodontic titanium alloy (TiAlV) implant covered with EPD coating containing chitosan and Titanium Nitride nanoparticles
TiNNPs/PLA	Orthodontic titanium alloy (TiAlV) implant covered with EPD coating containing chitosan and Titanium Nitride nanoparticles with additional PLA protective layer
BNPs	Orthodontic titanium alloy (TiAlV) implant covered with EPD coating containing chitosan and Boron Nitride particles
BNPs/PLA	Orthodontic titanium alloy (TiAlV) implant covered with EPD coating containing chitosan and Boron Nitride particles with additional PLA protective layer

**Table 2:** Antibacterial activity of Ti6Al4V substrate and EPD layers.

	Antibacterial Effectiveness	
Initial Colony Forming Units (CFU/mL): 1.5·10^5^	*S. aureus* ATCC 25923 CFU/mL	*E. coli* ATCC 25922 CFU/mL
Ti6Al4V	7.65·10^7^	7.82·10^6^
TiNPs	2.60·10^2^	5.00·10^3^
TiNNPs	9.60·10^5^	4.20·10^7^
BNPs	2.80·10^7^	1.93·10^6^

## Data Availability

The original contributions presented in this study are included in the article. Further inquiries can be directed to the corresponding authors.

## Supplementary Materials

The following supporting information can be downloaded at: https://www.mdpi.com/article/10.3390/jfb16090343/s1. Figure S1. SEM observations of TiNPs (a), STEM and TEM observation of TiNNPs (b), SEM observation of BNPs (c) powders. Figure S2. BET surface area analysis of TiNP (a), TiNNP (b), BNP (c) powders. Figure S3. BET surface area analysis of TiNP (a), TiNNP (b), BNP (c) powders. Figure S4. Particle Size distribution of TiNNP powders analyzed using Dynamic Light Scattering (DLS). Figure S5. Particle Size distribution of BNP powder analyzed using Dynamic Light Scattering (DLS).
