# Supplementary material for: Electrophoretic Coatings for Orthodontic Implants: Evaluation of Surface Properties, Adhesion, and Antibacterial Activity in Simulated Implantation Trials
Source: J Funct Biomater. 2025 Sep 12;16(9):343. doi: 10.3390/jfb16090343 (PMC12470570; doi:10.3390/jfb16090343)
Supplement: Supplementary file 1 [file jfb-16-00343-s001.zip › jfb-3789847-supplementary.pdf]

Supplementary Information

Electrophoretic Coatings for Orthodontic Implants: Evaluation of Surface Properties, Adhesion, and Antibacterial Activity in Simulated Implantation Trials

Maria Biegun-Żurowska [1,*], Karolina Klesiewicz [2], Katarzyna Matysiak [1], Marcin Gajek [1], Alicja Rapacz-Kmita [1] and Magdalena Ziąbka [1,*]

[1] Department of Ceramics and Refractories, Faculty of Materials Science and Ceramics, AGH University of Krakow, 30 Mickiewicza Av., 30-059 Krakow, Poland; kmatysiak@agh.edu.pl (K.M.); mgajek@agh.edu.pl (M.G.); kmita@agh.edu.pl (A.R.-K.)

[2] Department of Pharmaceutical Microbiology, Faculty of Pharmacy, Jagiellonian University Medical College, 9 Medyczna Str., 30-688 Krakow, Poland; karolina.klesiewicz@uj.edu.pl

* Correspondence: biegun@agh.edu.pl (M.B.-Ż.); ziabka@agh.edu.pl (M.Z.)

1. Powder characteristics

1.1. SEM observations

Figure S1 provides a detailed microscopic analysis of the powders, focusing on particle size and morphology. Panel (a) presents Scanning Electron Microscopy (SEM) images of the Titanium Nitride microparticles (TiNPs). The material has a habit of well-formed, solid microparticles with a range of sizes ranging from approximately 1.36 μm to 5.55 μm. At higher magnification, the distinct angular and polyhedral morphology of the particles becomes visible, characteristic of hard crystalline materials. In contrast, panel (b) shows significantly smaller nanoparticles of Titanium Nitride (TiNNPs) observed by Scanning Transmission Electron Microscopy (STEM) and Transmission Electron Microscopy (TEM). It can be seen that nanoparticles are quasi-spherical with a very uniform primary particle size ranging from 18.6 nm to 20.1 nm; however a strong tendency to form extended, chain-like agglomerates is also noticeable. Finally, panel (c) shows SEM images of Boron Nitride particles (BNPs) forming large agglomerates, which are composed of thin, nanosized, platelet-like particles, characteristic of hexagonal boron nitride. The lateral dimensions of these individual nanoplatelets vary, with measured sizes ranging from approximately 45 nm to over 100 nm.

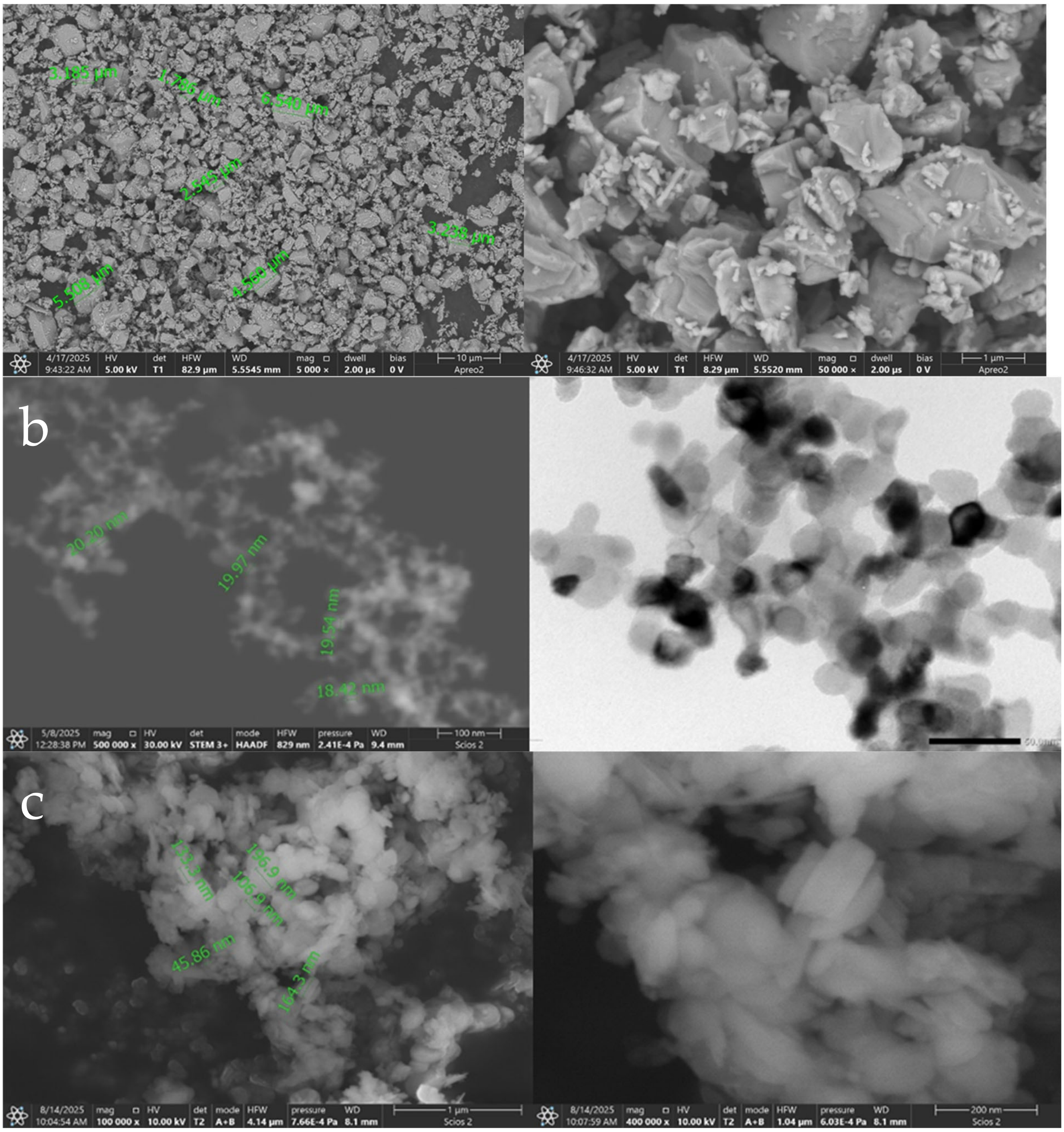

Figure S1. SEM observations of TiNPs (a) , STEM and TEM observation of TiNNPs (b), SEM observation of BNPs(c) powders.

1.2. BET surface area analysis

The specific surface area of the powders was determined by the Brunauer–Emmett–Teller (BET) method using a Micromeritics ASAP 2020 Plus analyzer. The measurements were based on nitrogen (N_2) physisorption at 77 K, and all samples were degassed under vacuum for 24 hours at 150°C prior to the analysis. The results showed a specific surface area of 2.33 m^2/g for titanium nitride microparticles (TiNPs, Figure S2 a) and a substantially higher value of 61.00 m^2/g for the corresponding TiNNPs (Figure S2 b). Boron nitride microparticles yielded a specific surface area of 39.72 m^2/g (Figure S2 c). The high linearity of the BET plots for all samples (correlation coefficient > 0.9999) confirms the reliability and accuracy of the measurements.

a

BET Report

BET surface area:	2,3281 ± 0,0013 m²/g
Slope:	41,37530 ± 0,02320 g/mmol
Y-intercept:	0,53054 ± 0,00318 g/mmol
C:	78,986835
Qm:	0,02386 mmol/g
Correlation coefficient:	0,9999995
Molecular cross-sectional area:	0,1620 nm²

b

BET Report

BET surface area:	61,0001 ± 0,1617 m²/g
Slope:	1,58244 ± 0,00420 g/mmol
Y-intercept:	0,01689 ± 0,00058 g/mmol
C:	94,697235
Qm:	0,62526 mmol/g
Correlation coefficient:	0,9999894
Molecular cross-sectional area:	0,1620 nm²

c

BET Report

BET surface area:	39.7189 ± 0.0342 m²/g
Slope:	2.44052 ± 0.00208 g/mmol
Y-intercept:	0.01572 ± 0.00039 g/mmol
C:	156.217041
Qm:	0.40713 mmol/g
Correlation coefficient:	0.9999982
Molecular cross-sectional area:	0.1620 nm²

Figure S2. BET surface area analysis of TiNP (a) , TiNNP (b), BNP(c) powders.

1.3. Particle size distribution

The particle size distribution of the Titanium Nitride microparticle (TiNP) powder was analyzed using a Malvern Mastersizer 2000 laser diffraction instrument equipped with a Hydro 2000S (A) wet dispersion unit. Water was used as the dispersant (Refractive Index, RI = 1.330), and a laser obscuration of 5.534% was maintained during the measurement. The "General purpose" analysis model, based on Mie scattering theory, was employed for the calculations, with the particle refractive index set to 1.350 and an absorption value of 1. The analysis revealed a unimodal particle size distribution with a volume-weighted mean diameter (D) of 2.815 μm and a surface-weighted mean diameter (D) of 1.348 μm. The median particle size (d(0.5)) was 1.471 μm, with the d(0.1) and d(0.9) percentiles determined to be 0.611 μm and 5.534 μm, respectively. The polydispersity of the powder is indicated by a Span of 1.987. The results show that the majority of the particle volume is concentrated within a range of approximately 0.2 μm to 10 μm (Figure S3).

Parameter	Value
Sample	TiNPs
d(0.1) (µm)	0.582
d(0.5) / Median (µm)	2.492
d(0.9) (µm)	5.534
Vol. Weighted Mean D (µm)	2.815
Span	1.987
d(0.1) (µm)	0.582

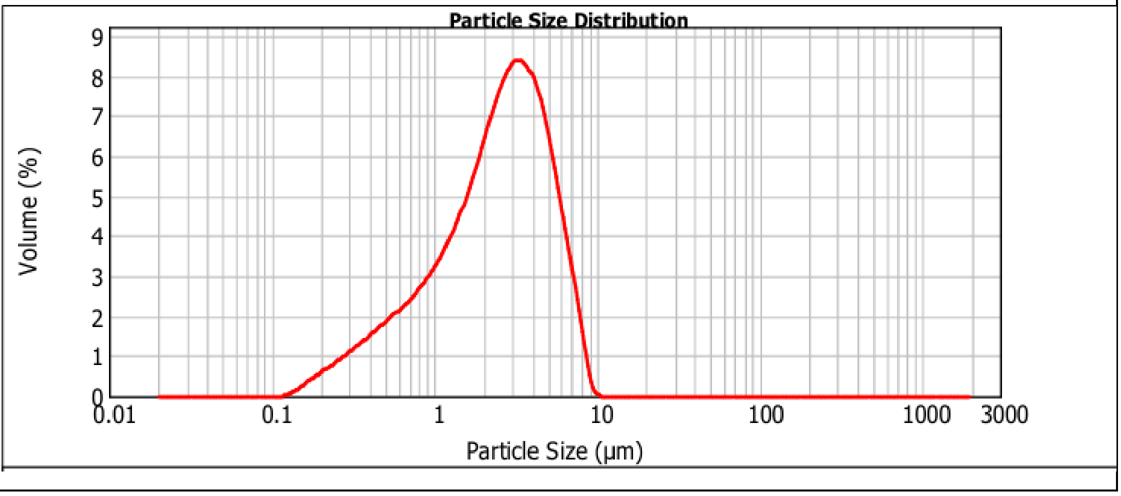

Figure S3. BET surface area analysis of TiNP (a) , TiNNP (b), BNP(c) powders.

The hydrodynamic diameter and size distribution of the TiNNP and BNP powders were determined using Dynamic Light Scattering (DLS). The analysis was conducted on a Malvern Panalytical Zetasizer instrument (Zetasizer Ver. 7.13). For the measurement, the particle samples were dispersed in water and transferred into a disposable sizing cuvette. The measurements were performed at a controlled temperature of 25.0°C. The DLS technique yields the intensity-weighted mean hydrodynamic diameter (Z-average) and the Polydispersity Index (PDI), which quantifies the breadth of the size distribution.

The analysis of the TiNNP sample revealed a Z-average hydrodynamic diameter of 177.6 nm. The sample exhibited a Polydispersity Index (PDI) of 0.128, which indicates a relatively uniform and monodisperse nanoparticle size distribution. The number-weighted size distribution shows a primary peak centered at 120.9 nm with a standard deviation of 43.72 nm. The result quality was determined to be good, confirming the reliability of the measurement (Figure S4).

Parameter	Value
Sample	TiNNPs
Z-Average	177.6 nm
Polydispersity Index (PDI)	0.128
Main Peak (Number Dist.)	120.9 nm
Standard Deviation for Peak	43.72 nm
Result Quality	Good

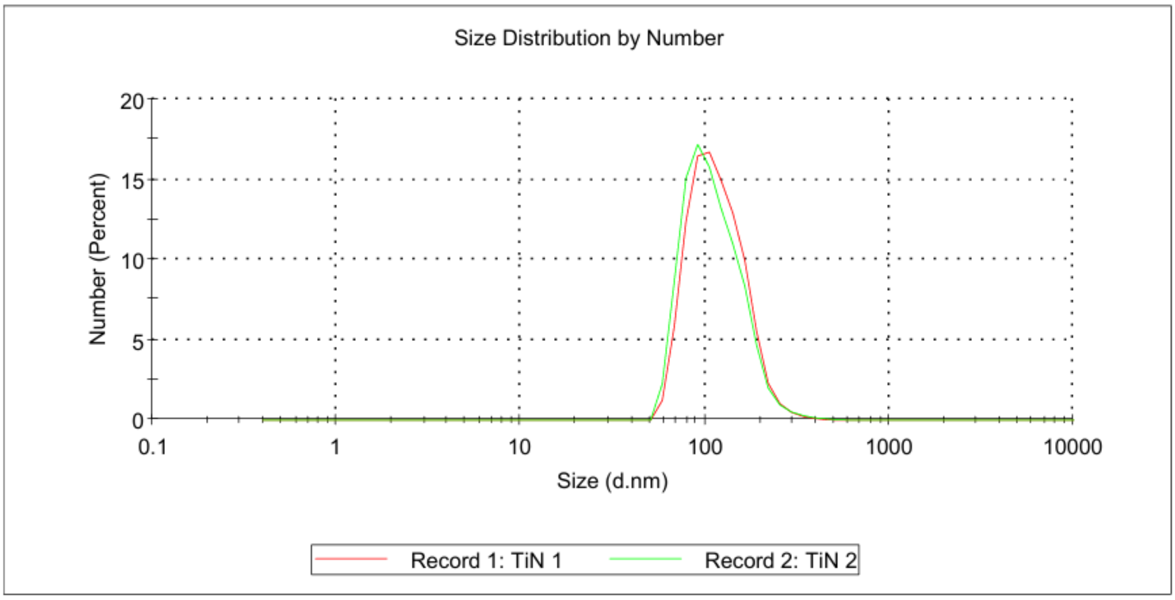

Figure S4. Particle Size distribution of TiNNP powders analyzed using Dynamic Light Scattering (DLS).

Subsequent characterization of the BNPs via Dynamic Light Scattering (DLS) determined the intensity-weighted mean hydrodynamic diameter (Z-Average) to be 230.3 nm. The material exhibited an exceptionally low Polydispersity Index (PDI) of 0.012, which is indicative of a highly homogeneous and monodisperse particle population. The corresponding number-weighted size distribution was unimodal, centered at 205.0 nm with a standard deviation of 51.59 nm. These data, for which the measurement quality was confirmed as good, are graphically represented in Figure S5.

Parameter	Value
Sample	BNPs
Z-Average	230.3 nm
Polydispersity Index (PDI)	0.012
Main Peak (Number Dist.)	205.0 nm
Standard Deviation for Peak	51.59 nm
Result Quality	Good

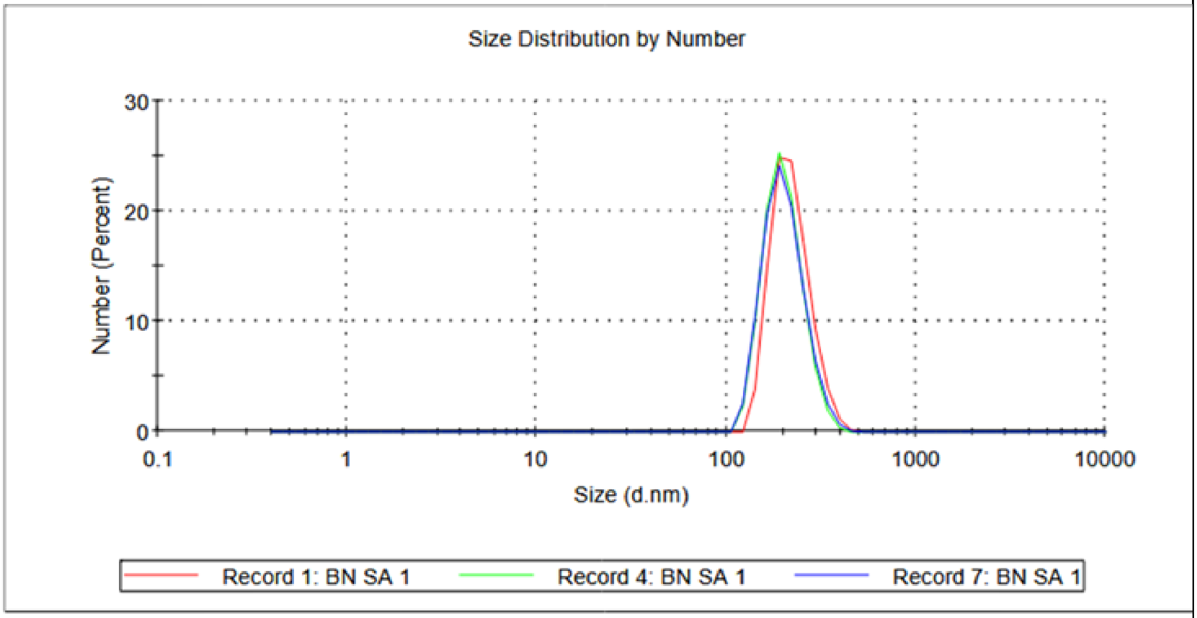

Figure S5. Particle Size distribution of BNP powder analyzed using Dynamic Light Scattering (DLS).
